# Lipoarabinomannan Decreases Galectin-9 Expression and Tumor Necrosis Factor Pathway in Macrophages Favoring *Mycobacterium tuberculosis* Intracellular Growth

**DOI:** 10.3389/fimmu.2017.01659

**Published:** 2017-11-27

**Authors:** Leslie Chávez-Galán, Lucero Ramon-Luing, Claudia Carranza, Irene Garcia, Isabel Sada-Ovalle

**Affiliations:** ^1^Laboratory of Integrative Immunology, National Institute of Respiratory Diseases Ismael Cosio Villegas, Mexico City, Mexico; ^2^Department of Microbiology, National Institute of Respiratory Diseases Ismael Cosio Villegas, Mexico City, Mexico; ^3^Department of Pathology and Immunology, Centre Medical Universitaire, Faculty of Medicine, University of Geneva, Geneva, Switzerland

**Keywords:** macrophage, *Mycobacterium tuberculosis*, lipoarabinomannan, galectin-9, tumor necrosis factor pathway

## Abstract

Lipoarabinomannan (LAM) is a lipid virulent factor secreted by *Mycobacterium tuberculosis* (*Mtb*). LAM can be found in the sputum and urine of patients with active tuberculosis. When human monocytes are differentiated into macrophages [monocyte-derived macrophages (MDM)] in the presence of LAM, MDM are poorly functional which may limit the immune response to *Mtb* infection. Our previous studies have shown that TIM3 and galectin (GAL)9 interaction induces anti-mycobacterial activity, and the expression levels of TIM3 and GAL9 are downregulated during *Mtb* infection. We postulated that LAM affects GAL9/TIM3 pathway, and, in consequence, the ability of the macrophage to control bacterial growth could be affected. In this work, we have generated MDM in the presence of LAM and observed that the expression of TIM3 was not affected; in contrast, GAL9 expression was downregulated at the transcriptional and protein levels. We observed that the cell surface and the soluble form of tumor necrosis factor (TNF) receptor 2 were decreased. We also found that when LAM-exposed MDM were activated with LPS, they produced less TNF, and the transcription factor proteinase-activated receptor-2 (PAR2), which is involved in host immune responses to infection, was not induced. Our data show that LAM-exposed MDM were deficient in the control of intracellular growth of *Mtb*. In conclusion, LAM-exposed MDM leads to MDM with impaired intracellular signal activation affecting GAL9, TNF, and PAR2 pathways, which are important to restrict *Mtb* growth.

## Introduction

*Mycobacterium tuberculosis* (*Mtb*) is the infectious agent of tuberculosis (TB), which is one of the leading causes of morbidity and mortality around the world. World Health Organization reported that in 2014, there were 9 million new cases of TB and 1.5 million deaths globally (1).

*Mycobacterium tuberculosis* is an intracellular pathogen whose cell wall has low permeability, which confers resistance to therapeutic agents. *Mtb* cell wall is rich in lipids (30–60% of dry weight) that are strategically located and contribute to its virulence (2). Lipoarabinomannan (LAM) is a major lipoglycan found in the mycobacterial cell wall. Slow growing mycobacteria, as *Mtb*, have a LAM that structurally possess mannosyl caps (hereafter LAM), which is constantly released from the cell wall of *Mtb* (3). LAM is considered as an important virulence factor that can be identified in the sputum and urine of patients with TB (TB patients). In fact, LAM has an amphiphilic nature favoring its association with host lipid carriers, which suggests that peripheral blood mononuclear cells (PMBCs) from patients with TB might be exposed to LAM during the natural history of TB (4, 5). Consequently, LAM could be employed as a biomarker to predict outcomes during anti-TB therapy.

Macrophage is a professional antigen-presenting cell that has as origin the monocyte and plays a critical role in the pathogenesis of TB. It has been described that *Mtb* downregulates the macrophage activation in order to facilitate their persistence (6). In particular, LAM has been studied for its immunomodulatory properties in macrophage (7, 8). Using an *in vitro* model where monocytes were stimulated with LAM, we have shown that LAM exposure influenced monocyte differentiation generating poorly functional macrophage, a phenomenon that could limit the quality of immune response to *Mtb* infection (9).

The molecule T-cell immunoglobulin and mucin domain 3 (TIM3) is a receptor initially identified as a specific marker for Th1-type immune cells. Nowadays, there is evidence that TIM3 is expressed in diverse myeloid and lymphoid cells and that its failure or absence is associated with the development of several diseases including infectious diseases (10). The interaction of TIM3 with galectin (GAL)9, which is one of the TIM3 ligands, has been shown to induce macrophage activation for bactericidal functions and to control TB infection using a murine model of *Mtb* infection (11). However, TB patients have a lower frequency of CD14^+^TIM3^+^ cells in peripheral blood, suggesting that this downregulation could be a mechanism to disturb the immune response in the host (12).

*Mycobacterium tuberculosis* infection activates cellular pathways in macrophages to initiate immune response, which involves the production of cytokines playing a critical role to regulate host defense. Tumor necrosis factor (TNF) is a pro-inflammatory cytokine, which orchestrates a wide range of functions. It is synthesized as a transmembrane TNF (tmTNF) that is proteolytically processed by the metalloprotease TNF-α-converting enzyme (TACE) to generate the soluble TNF (solTNF). Both tmTNF and solTNF isoforms exert their bioactivities via binding of two different receptors, TNF receptor 1 (TNFR1) and TNF receptor 2 (TNFR2) (13). In animal models, TNF pathway has been shown to be crucial for the activation of host protective immune responses against mycobacterial infection (14, 15). In human, TNF inhibition, used for the treatment of autoimmune-inflammatory diseases, has been associated with an increased risk of opportunistic infections including TB (16, 17).

Considering the reported information on the relevance of TIM3/GAL9 pathway in the defense against mycobacterial infections, it is still not clear whether LAM may modify TIM3/GAL9 pathway and impair host defense mechanisms. In this study, we explore possible modifications of TIM3/GAL9 pathway due to LAM exposure that could reduce macrophage responses and compromise bacterial elimination. We have characterized the expression profile of TIM3 and GAL9 on human monocyte-derived macrophages (MDM) exposed to LAM during their differentiation process. We have evaluated the capacity of these MDM to produce cytokines, to activate transcription factors, and to control *Mtb* growth. Our findings indicate that LAM exposure induces MDM with reduced GAL9 expression, which in response to an activation stimulus leads to weak cytokine and intracellular signals affecting the control of *Mtb* survival.

## Materials and Methods

### Ethics Statement

Peripheral blood mononuclear cells were obtained from buffy coats by the blood bank at the National Institute of Respiratory Diseases Ismael Cosio Villegas, Mexico City. The study was approved by the Institutional Review Board (IRB# B04-12) and was conducted following the principles stipulated in the Declaration of Helsinki.

### LAM from *Mtb*-H37Rv

Purified LAM was obtained from Colorado State University (NR-14848). The lipid was then reconstituted in distilled water as recommended.

### Preparation of PBMC and Magnetic Cell Sorting

Peripheral blood mononuclear cells were isolated from buffy coats by standard Lymphoprep™ (Accurate Chemical-Scientific, Westbury, NY, USA) gradient centrifugation. Monocytes were isolated by positive selection using anti-CD14-coated magnetic microbeads (Miltenyi Biotec). Enrichment of the CD14^+^ fraction was routinely >95%, as analyzed by flow cytometry. CD14^+^ cells were plated at 1 × 10^6^ cells/well in 24-well plates (Costar, ON, Canada) with RPMI 1640 medium (GIBCO, Grand Island, NY, USA), supplemented with l-glutamine (2 mM; GIBCO, Grand Island, NY, USA), streptomycin, penicillin, and 10% heat-inactivated fetal bovine serum (GIBCO, Grand Island, NY, USA). CD14^+^ cells were cultured for 7 days at 37°C in a humidified atmosphere containing 5% CO_2_. After 7 days, viable cells were considered to be MDM based on their expression profile of differentiation molecules as previously reported (9).

### Differentiation and Stimulation of MDM with LAM

**Diagram 1:**
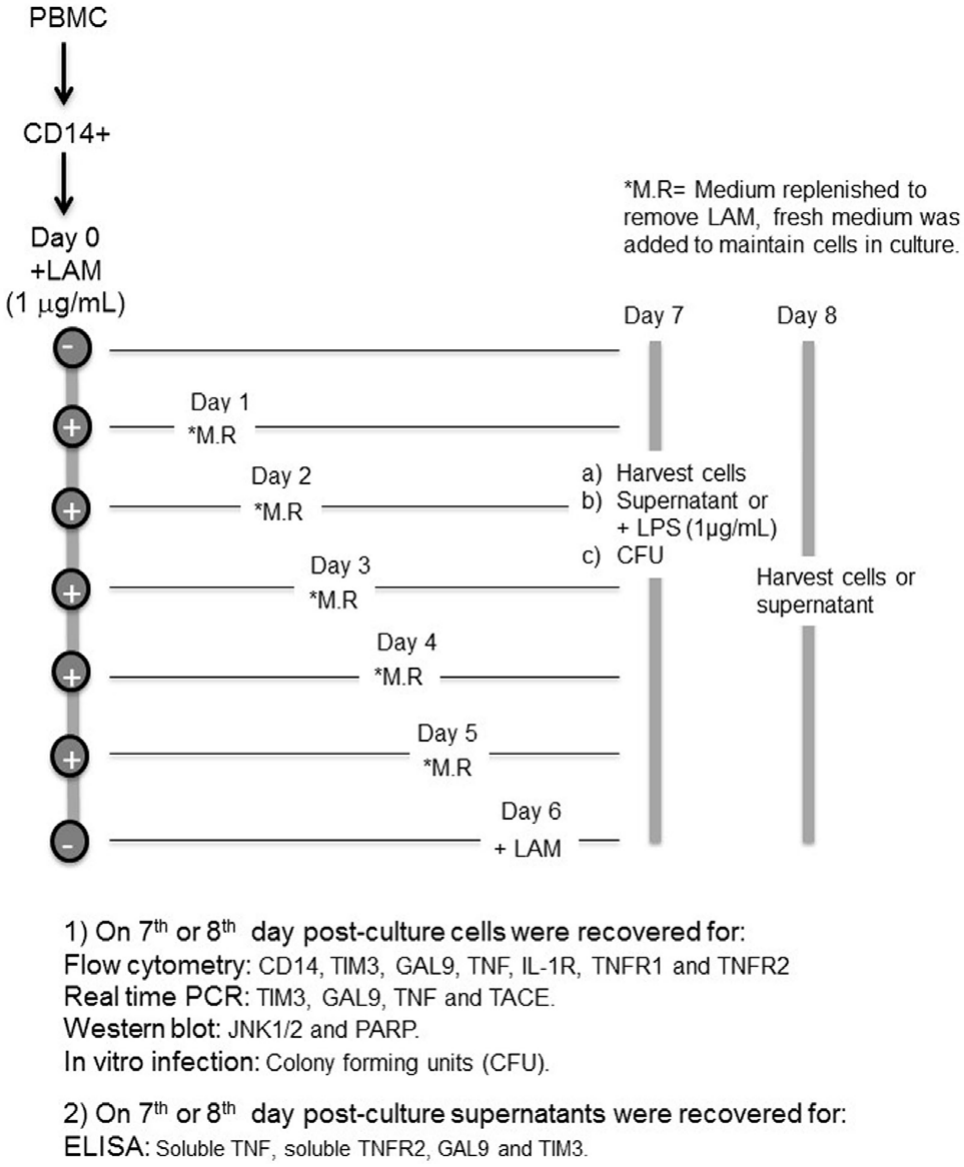
Design of the experimental strategy.

CD14^+^ cells were cultured to differentiate into MDM in the presence of LAM using an *in vitro* model that we have previously reported (9). Briefly, CD14^+^ (1 × 10^6^) cells were seeded in a 24-well plate and stimulated or not with LAM (1 µg/mL), for 1, 2, 3, 4, or 5 days. Every day, culture medium was replaced by medium without LAM and cells recovered at the 7th or 8th day of culture. Two control conditions were used: first, medium without LAM during the 7 or 8 days of culture and, second, cells were left for differentiation without LAM for 6 days, and then, LAM was added and maintained until day 7 or 8 of culture. At day 7 of culture, we established two different protocols, the first consisted in recovering cells for FACS analysis, RNA extraction (real-time PCR), cellular lysis (western blot assays), for *in vitro* infection and mycobacterial quantification [colony-forming units (CFUs)], and the supernatants were for ELISA. In the second protocol, LPS (1 µg/mL) was added at day 7 for 24 h (day 8) to perform RNA extraction (real-time PCR) and the supernatants recovered for ELISA (Diagram 1).

### Flow Cytometry

Cells were stained for 20 min at 4°C with fluorochrome-conjugated mAb against CD14, TIM3, GAL9, TNF, and interleukin-1 receptor (IL-1R) (BioLegend, San Diego, CA, USA). Both TNFR1 and TNFR2 (R&D Systems, Minneapolis, MN, USA). After incubation, cells were washed and re-suspended in staining buffer (BD Biosciences, San Jose, CA, USA) prior to FACS analysis. Data were collected using a FACS Aria II flow cytometer (Becton Dickinson, San Jose, CA, USA) and FACS Diva software (V.8.01). Cells were then analyzed with FlowJo (Tree Star, Inc., Ashland, OR, USA). Typically, 20,000 events were acquired.

### Western Blot

After incubation, cells were washed twice with PBS and lysed in Laemmli buffer. Equal amounts of protein were subjected to Mini-protean TGK 4–15% gels and transferred to a 0.2-μm pore size Trans-Blot Turbo TM PVDF membrane (Bio-Rad Laboratories, Hercules, CA, USA). Western blot was performed using the following antibodies: c-Jun N-terminal kinase (JNK) 1/2 and poly(ADP-ribose) polymerase (PARP) (R&D Systems). Protein bands were detected by incubating with horseradish peroxidase-labeled antibodies and developed with enhanced chemoluminiscence reagent (Thermo Scientific, Pierce Biotech., Rockford, IL, USA) and ChemiDoc MP Imaging System (Bio-Rad). Band densities were analyzed by densitometry using online IMAGEJ software provided by the NIH (http://rsb.info.nih.gov/ij/index.html), as described by Luke Miller (http://www.lukemiller.org/journal/2007/08/quantifying-western-blots-without.html). Each sample was normalized using glyceraldehyde 3-phosphate dehydrogenase as a loading control.

### Real-time PCR for TIM3, GAL9, TNF, TACE, RAB33A and Proteinase-Activated Receptor-2 (PAR2) Gene Expression

For the analysis of TIM3, GAL9, TNF, TACE, RAB33A, and PAR2 gene expressions, MDM exposed to LAM were generated as described in the previous section. Total RNA from 1 × 10^6^ cells was isolated using RNeasy Mini Kit (Qiagen, Hilden, Germany) following the manufacturer’s protocol. DNA genome was eliminated with RNase-Free DNase Set (Qiagen). RNA was eluted in 30 µL of nuclease-free water and the quantity of extracted RNA was evaluated by Qubit™ assay kit with the Qubit 2.0 Fluorometer (Life Technologies, Carlsbad, CA, USA). A total of 67.5 ng of total RNA was converted to cDNA using High Capacity cDNA Reverse Transcription Kit (Applied Biosystems, Foster City, CA, USA) as per the manufacturer’s guidelines. Gene expression analyses was performed with the StepOnePlus™ Real-Time PCR Systems (Applied Biosystems) using standard thermal cycling conditions and Taqman assays specific for TIM3 (Hs00958618_m1), GAL9 (Hs01088490_m1), TNF (Hs01113624_g1), TACE (Hs01041915_m1), RAB33A (Hs00191243_m1), and PAR2 (Hs00608346_m1). Data were normalized to two endogenous controls, ACTB (β-actin) (Hs01060665_g1) and 18S (18S ribosomal RNA gene) (Hs03928990_g1). Before gene expression analysis, cDNA samples were serially diluted to 1:5 or 1:2 and 2.5 µL were used as template for the quantitative real-time PCR (qPCR) to perform the validation of the delta-delta CT method. Same cDNA dilutions were used for the all qPCR assays, and relative gene expression values of all different gene targets were calculated using the 2-DDCT formula. The expression of each target gene is presented as the “fold change” relative to that of control condition (MDM without LAM). All the qPCRs were run in duplicate along with no-template controls.

### ELISA

Culture supernatants from MDM exposed to LAM (±LPS) were recovered and stored at −80°C for future analysis. We used the standard sandwich ELISA for GAL9, TIM3, and TNFR2 (R&D Systems) and TNF (BioLegend, Inc.) following the manufacturer’s instruction. All proteins in the culture supernatants were quantified by comparison with the appropriated recombinant standard.

### Bacteria

*Mycobacterium tuberculosis* strain H37Ra (*Mtb*-H37Ra) (ATCC-25177) was grown for 21 days in Middlebrook 7H9 broth. Mycobacteria were harvested and clumps were disrupted with sterile 3 mm glass beads and vortexed for 5 min. The concentration of disaggregated mycobacteria was determined by counting CFUs on 7H10 agar plates in triplicate serial dilutions and after 21 days of incubation. Bacterial stock was stored at −70°C until use.

### Intracellular Growth of *Mtb*

To evaluate mycobacterial intracellular growth, MDM were plated in duplicated wells at a concentration of 2 × 10^5^ cells/well 96-well plates. MDM were infected with H37Ra at MOI of 1. After 1 h infection at 37°C in 5% CO_2_ incubator, plates were washed three times to remove any extracellular mycobacteria. Cells were lysed with 0.1% sodium dodecyl sulfate in 7H9 Milddlebrook medium and neutralized with 20% bovine serum albumin on days 0 (CFU_day 0_), 3 (CFU_day 3_), and 7 (CFU_day 7_) post-infection. Serial dilutions of the lysates from duplicate wells were plated on Middlebrook 7H10 agar by triplicate and CFU was determined after 21 days of incubation at 37°C and 5% CO_2_. Average CFU numbers were from duplicate assessments to obtain the percentage of change. % CFU was calculated as follows: CFU_day 0_ number per condition was considered as 100%, posteriorly the CFU number at day 3 or day 7 was obtained and calculated the percentage in relation to CFU_day 0_ from each individual condition experiment.

### Statistical Analysis

Data are shown as median ± interquartile range or median ± SD. Mann–Whitney *U* test was used to compare two groups, and a Kruskal–Wallis test with Dunn’s *post hoc* test when more than two groups were compared. Values of *P* < .05 were considered statistically significant (GraphPad Software, Inc., San Diego, CA, USA).

## Results

### TIM3 Expression in Macrophages Is Not Affected When Monocytes Are Exposed to LAM

Our previous results have shown that monocyte exposure to LAM promotes their differentiation into immature macrophages (9). This differentiation is not associated with an increased in cell death of MDM generated under LAM exposure compared to unexposed cells (Figure S1 in Supplementary Material). During MDM differentiation process, a low percentage of MDM lose or reduce CD14 expression (18). As differentiation markers can vary during MDM differentiation process, in this article, we are reporting the expression of TIM3 and other molecules on the total MDM population comprising CD14^−^ and CD14^+^ MDM and only on CD14^+^ MDM subpopulations.

**Figure 1:**
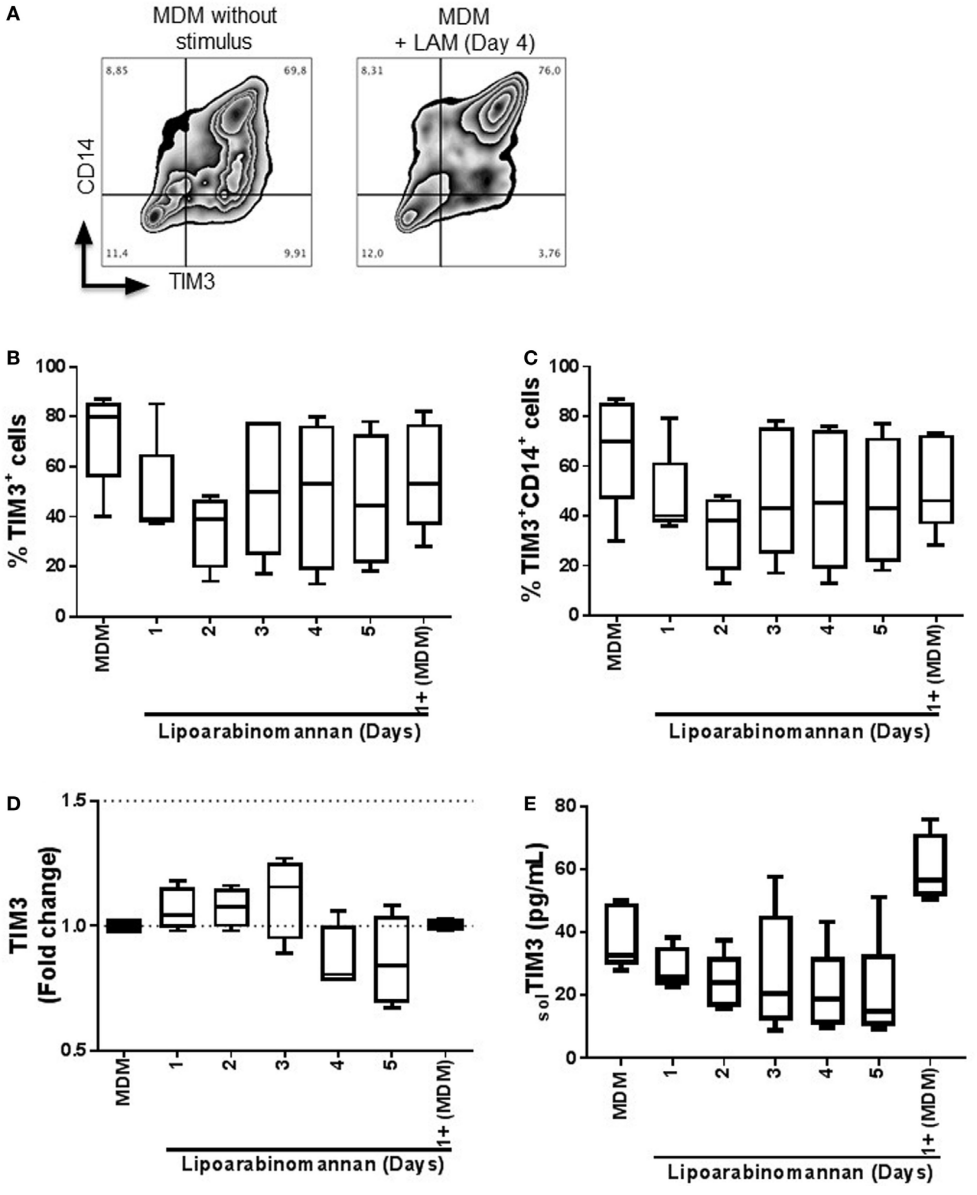
Expression and frequency of TIM3^+^ MDM is not modified when monocytes are exposed to LAM. Pure monocytes were exposed to LAM for 1, 2 3, 4, or 5 days and at day 7 cells were recovered to perform flow cytometry or qPCR. Flow cytometry representative zebra plot of MDM without stimulus or exposed to LAM for 4 days (left and right, respectively) **(A)**. Expression of TIM3 was analyzed by flow cytometry independent or dependent of CD14 co-expression (**B** and **C**). TIM3 relative gene expression was measured by real-time PCR **(D)**. Soluble form of TIM3 was measured in supernatant by ELISA **(E)**. Data are representative of five independent experiments. Box plot indicates median ± IQR (5-95). Kruskal-Wallis and Dunn *post-hoc* tests compared to unexposed MDM.

To determine if TIM3 expression is affected by the exposure of monocyte to LAM, we measured TIM3 expression on MDM that did not reveal changes of the percentage and intensity of TIM3^+^ MDM (Figure 1A) (data not shown). Total MDM or CD14^+^ MDM derived from monocytes exposed to LAM for 2 days showed a decrease of 50% compared to unexposed MDM, but the difference was not statically significant (Figures 1B,C). In order to evaluate LAM effect on TIM3 expression at the transcription level, we performed gene expression analysis by real-time PCR and observed that TIM3 gene expression was similar for the different MDM groups, independently of LAM exposure time (Figure 1D). Finally, the level of soluble TIM3 was measured by ELISA and protein levels correlated with gene expression (Figure 1E). These data indicate that previous monocyte exposure to LAM generates MDM with a normal expression of TIM3.

### GAL9 Expression in Mature Macrophages Is Altered When Monocytes Are Exposed to LAM

**Figure 2:**
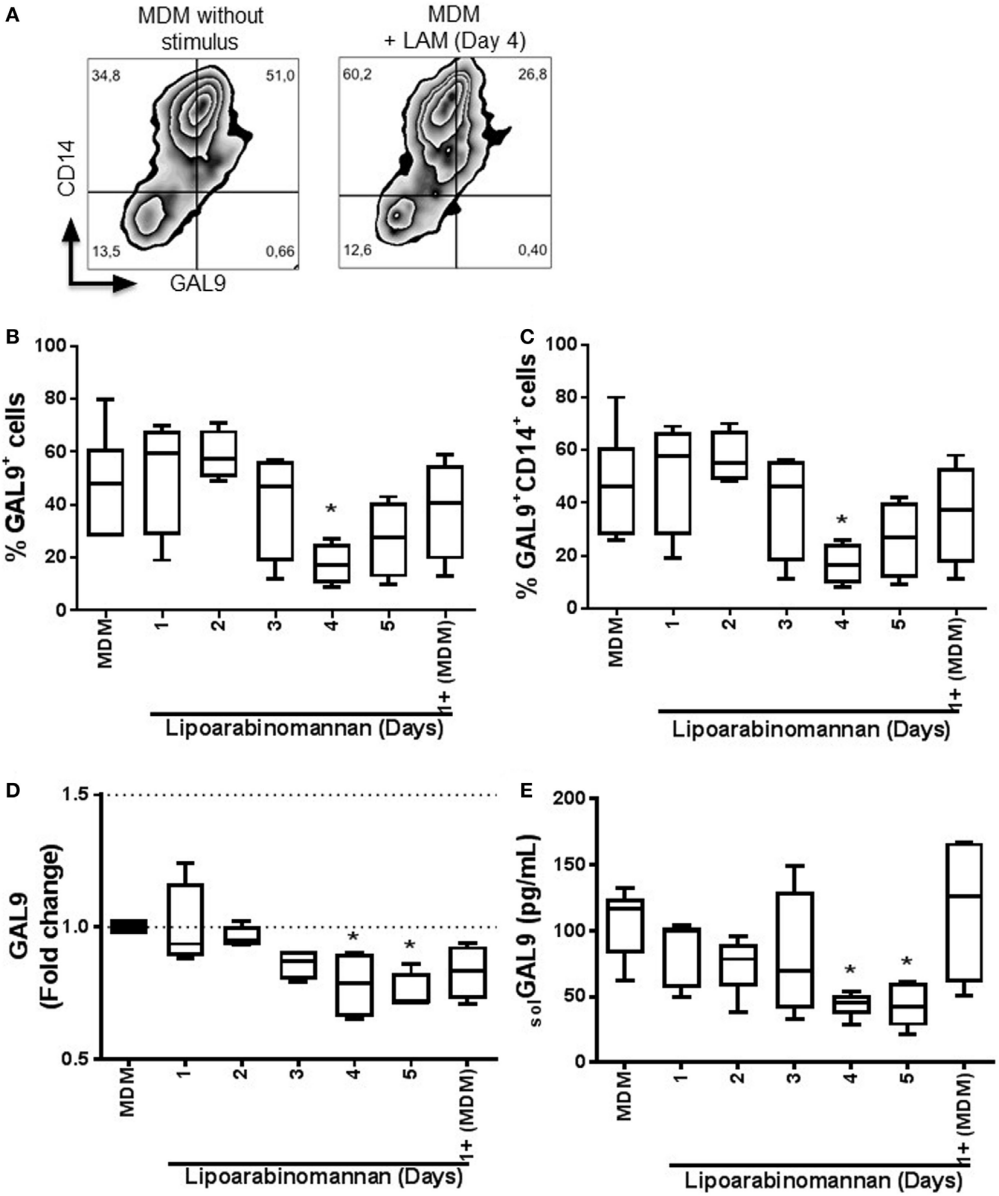
Expression and frequency of GAL9^+^ monocyte-derived macrophages (MDM) decrease when monocytes are exposed to lipoarabinomannan (LAM). Pure monocytes were exposed to LAM for 1, 2, 3, 4, or 5 days, and at day 7, cells and supernatants were recovered to perform flow cytometry, qPCR, and ELISA. Representative zebra plot of MDM without stimulus or exposed to LAM for 4 days (left and right, respectively) **(A)**. Flow cytometry analysis of the expression of galectin (GAL)9 independent or dependent of CD14 co-expression **(B,C)**. GAL9 relative gene expression was evaluated by real-time PCR **(D)**. Soluble form of GAL9 was measured in supernatant by ELISA **(E)**. Data are representative of five independent experiments. Box plot indicates median ± interquartile range (5–95). *P < 0.05, Kruskal–Wallis and Dunn’s *post hoc* tests compared to unexposed MDM.

Next, we address the question of whether LAM stimulus modulates the surface expression of GAL9, the ligand of TIM3. Flow cytometry analyses showed that when monocytes were exposed to LAM for 4 days, the frequency of GAL9^+^ MDM was reduced (Figure 2A). This lower frequency was independent of coexpresion with CD14 molecule on MDM surface (Figures 2B,C). To further define this effect, we evaluated GAL9 gene expression. Interestingly, we observed a significant decrease of GAL9 gene expression in MDM when monocytes were exposed to LAM. In MDM generated after 4 and 5 days of LAM exposure, GAL9 was decreased of 25 and 30%, respectively, in comparison to MDM without LAM stimulus (Figure 2D). Finally, the level of soluble GAL9 was measured by ELISA and protein levels correlated with gene expression (Figure 2E). These findings suggest that monocytes exposed during a long time (4–5 days) to LAM stimulus generated MDM exhibiting modified GAL9 expression.

### IL-1R Expression Is Not Affected in Mature Macrophages When Monocytes Are Exposed to LAM

**Figure 3:**
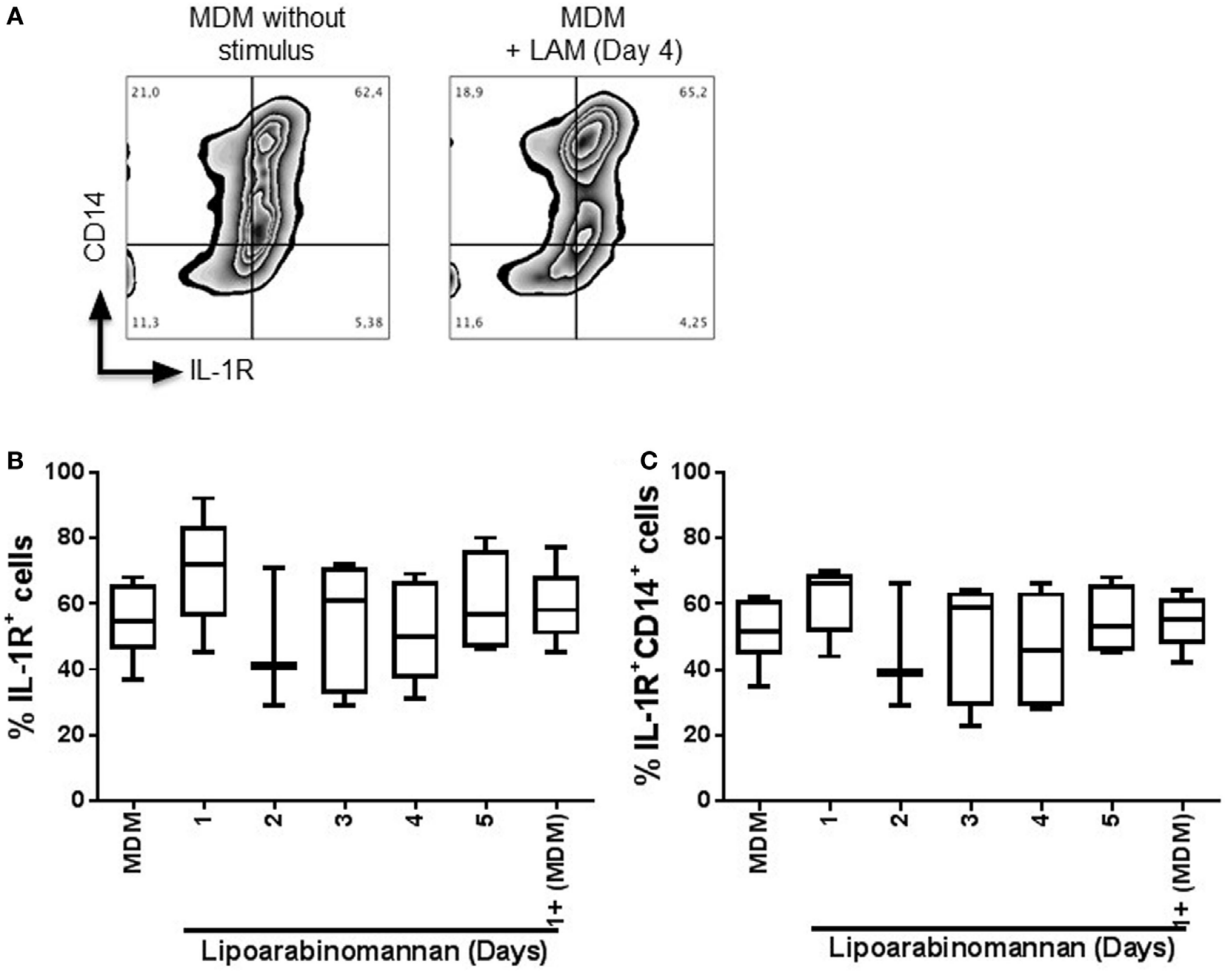
Frequency of interleukin-1 receptor (IL-1R^+^) monocyte-derived macrophages (MDM) is not modified when monocytes are exposed to lipoarabinomannan (LAM). Pure monocytes were exposed to LAM for 1, 2, 3, 4, or 5, and at day 7, cells were recovered. Flow cytometry representative zebra plot of MDM without stimulus or exposed to LAM for 4 days (left and right, respectively) **(A)**. Analysis of IL-1R^+^ MDM was done independent or dependent of CD14 co-expression **(B,C)**. Data are representative of 3–5 independent experiments. Box plot indicates median ± interquartile range (5–95). Kruskal–Wallis and Dunn’s *post hoc* tests compared to unexposed MDM.

Previously, it has been shown that the autocrine proinflamatory cytokine IL-1β signaling via IL-1R is an important mechanism by which TIM3–GAL9 interaction induces control of *Mtb* replication (11). To analyze if GAL9 downregulation observed after LAM monocyte exposure affects IL-1R pathway, we evaluated IL-1R expression on the cell surface of MDM generated under LAM stimulus (Figure 3A). Our flow cytometry data showed that LAM stimulus did not affect IL-1R expression on MDM as the frequency of MDM IL-1R^+^ was similar in MDM generated under different times of LAM exposure, and it was similar independently of co-expression with CD14 (Figures 3B,C). This suggests that even if LAM affects GAL9 expression on MDM, the IL-1β pathway dependent on IL-1R is probably not affected by LAM exposure.

### TNF Is Regulated in MDM When Monocytes Are Exposed to LAM, but They Are Unable to Produce TNF after LPS Activation

**Figure 4:**
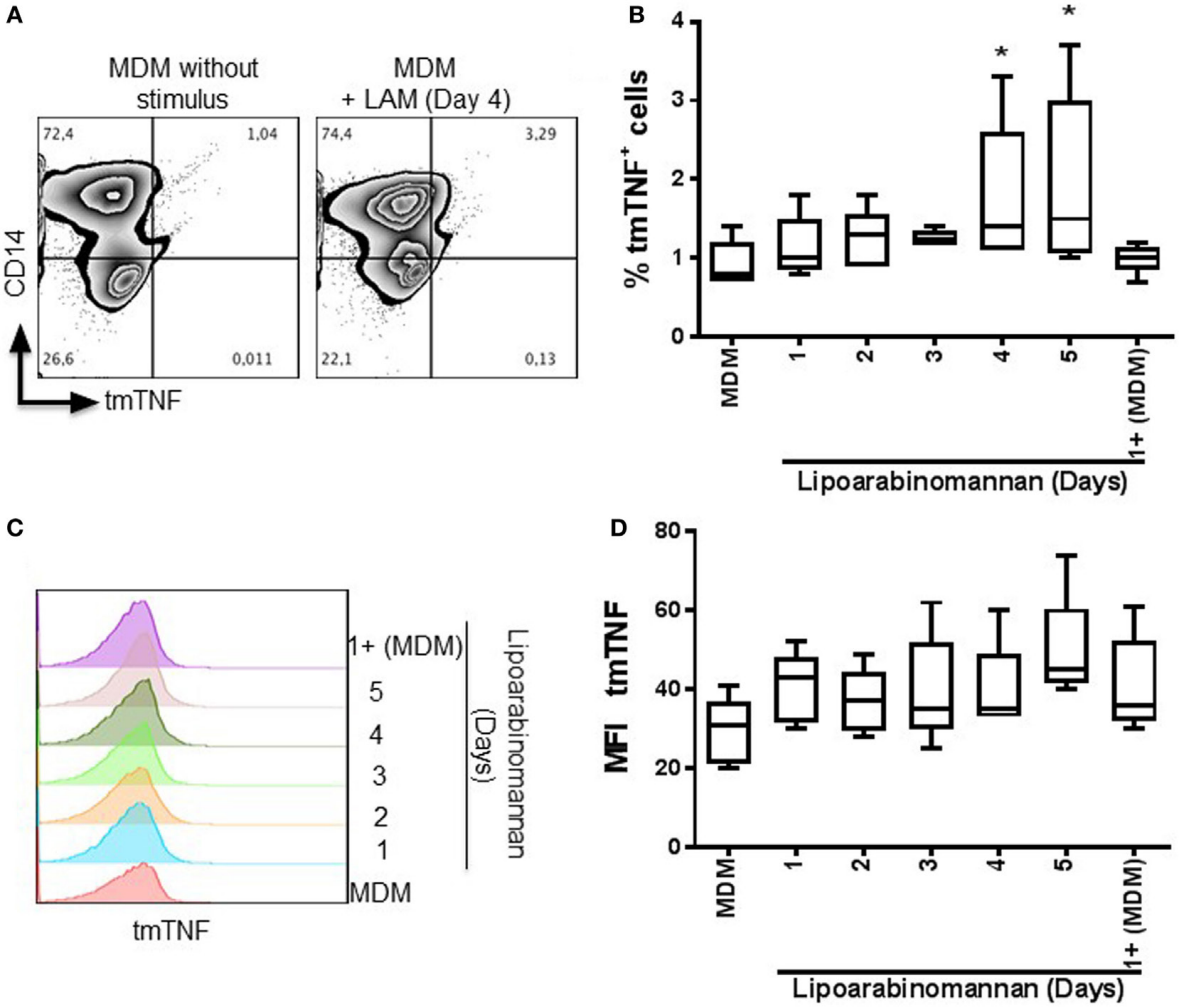
Transmembrane tumor necrosis factor (tmTNF) is increased when monocytes are exposed to lipoarabinomannan (LAM). Pure monocytes were exposed to LAM for 1, 2, 3, 4, or 5 days, and at day 7, cells were analyzed for tmTNF and CD14 expression by flow cytometry. Representative zebra plot of monocyte-derived macrophages (MDM) without stimulus or exposed to LAM for 4 days (left and right, respectively) **(A)**. Analysis of the percentage of tmTNF^+^ cells **(B)**. Histogram displays the median fluorescence intensity of tmTNF expression and its respective analysis [**(C,D)**, respectively]. Data are representative of four independent experiments. Box plot indicates median ± interquartile range (5–95). *P < 0.05, Kruskal–Wallis and Dunn’s *post hoc* tests compared to unexposed MDM.

It has been reported that secretion of IL-1β induced by TIM3–GAL9 interaction increased TNF signaling through the upregulation of TNF secretion and TNFR1 cell surface expression, which improved caspase-dependent restriction of intracellular *Mtb* growth (19). In contrast, we have reported that MDM generated under LAM stimulus for 3–5 days are less able to release solTNF after LPS stimulus (9). MDM expressing tmTNF form were measured by flow cytometry (Figures 4A–C). The frequency and the mean intensity of tmTNF^+^ MDM were increased in MDM generated under LAM stimulus for 4–5 days (Figures 4B,D).

**Figure 5:**
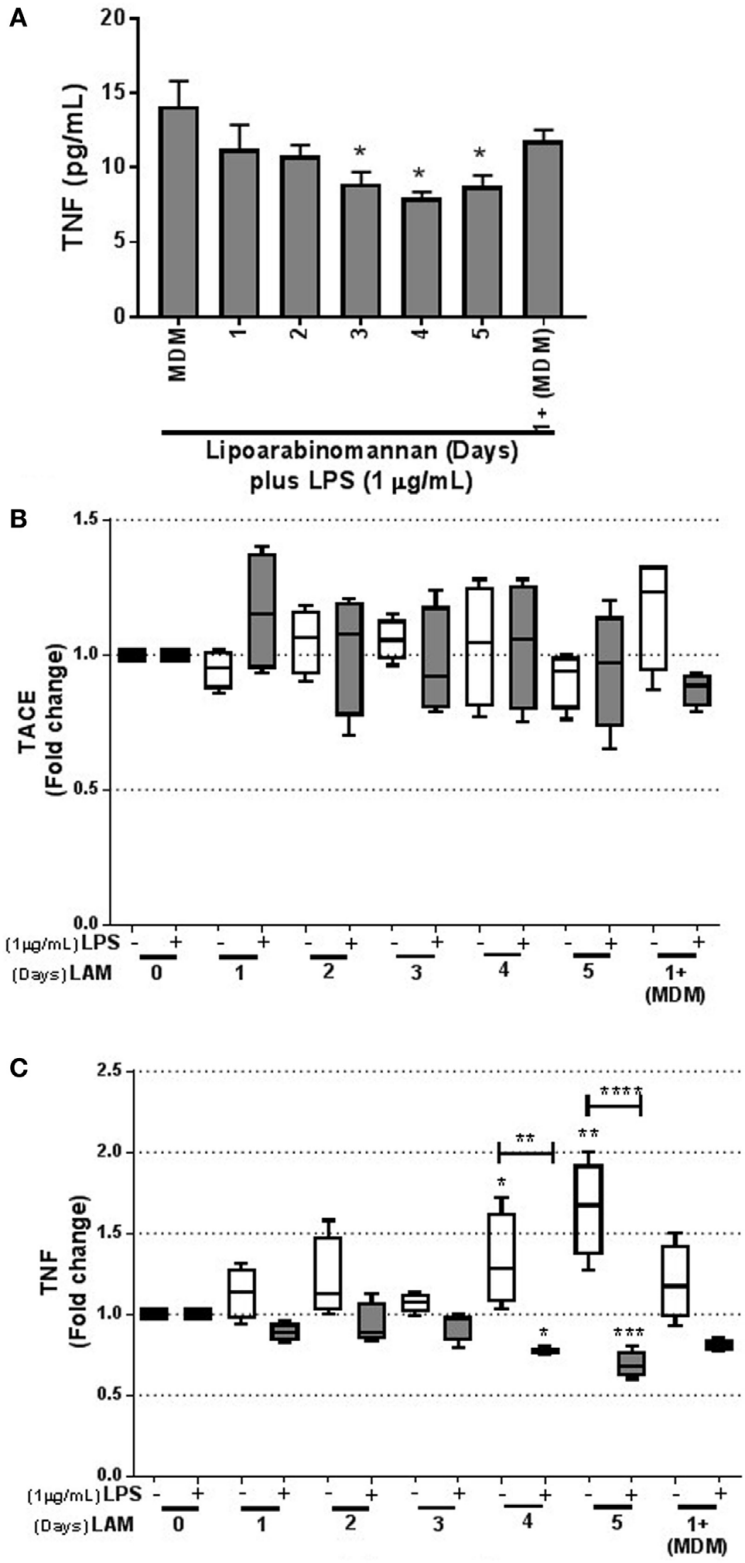
Tumor necrosis factor (TNF) synthesis is downregulated following LPS activation when monocytes are previously exposed to lipoarabinomannan (LAM). Pure monocytes were exposed to LAM for 1, 2, 3, 4, or 5 days, and at day 7, cells were stimulated or not with LPS (1 µg/mL) for 24 h and recovered at day 8 for RT-PCR. Soluble TNF in supernatant was measured by ELISA **(A)**. TACE and TNF relative expression was measured by real-time PCR [**(B,C)**, respectively]. Data are representative of 4–5 independent experiments. Box plot indicates median ± interquartile range (5–95), and bars represent median ± SD. *P < 0.05, **P < 0.01, ***P < 0.001, ****P < 0.0001. Kruskal–Wallis and Dunn’s *post hoc* tests. Monocyte-derived macrophages (MDM) exposed to LAM (white box) were compared with unexposed MDM, and MDM exposed to LAM plus LPS (gray box) were compared with unexposed MDM plus LPS.

To verify the inability of MDM exposed to LAM (3–5 days) to produce solTNF in response to LPS stimulus, we measured solTNF by ELISA and observed a downregulation of solTNF, confirming our previous reported data (9) (Figure 5A). To clarify this point, we measured TACE at the transcriptional level. Our data showed that TACE gene expression was not changed in MDM generated under LAM stimulus and also after a LPS stimulus (Figure 5B).

However, in concordance with the Figures 4 and 5A, TNF gene expression, measured also by qPCR, was increased in MDM derived from LAM exposed monocytes (for 4–5 days); but when MDM were activated with LPS, TNF gene expression was decreased (Figure 5C), as previously reported (9). Together, these data demonstrate that TNF is activated in response to LAM stimulus. However, mature MDM derived from monocytes exposed to LAM (4–5 days) were impaired in the capacity to produce TNF when activated with LPS, suggesting that these MDM are not able to efficiently activate intracellular pathways.

### TNFR2 but No TNFR1 Is Downregulated in MDM Exposed to LAM

**Figure 6:**
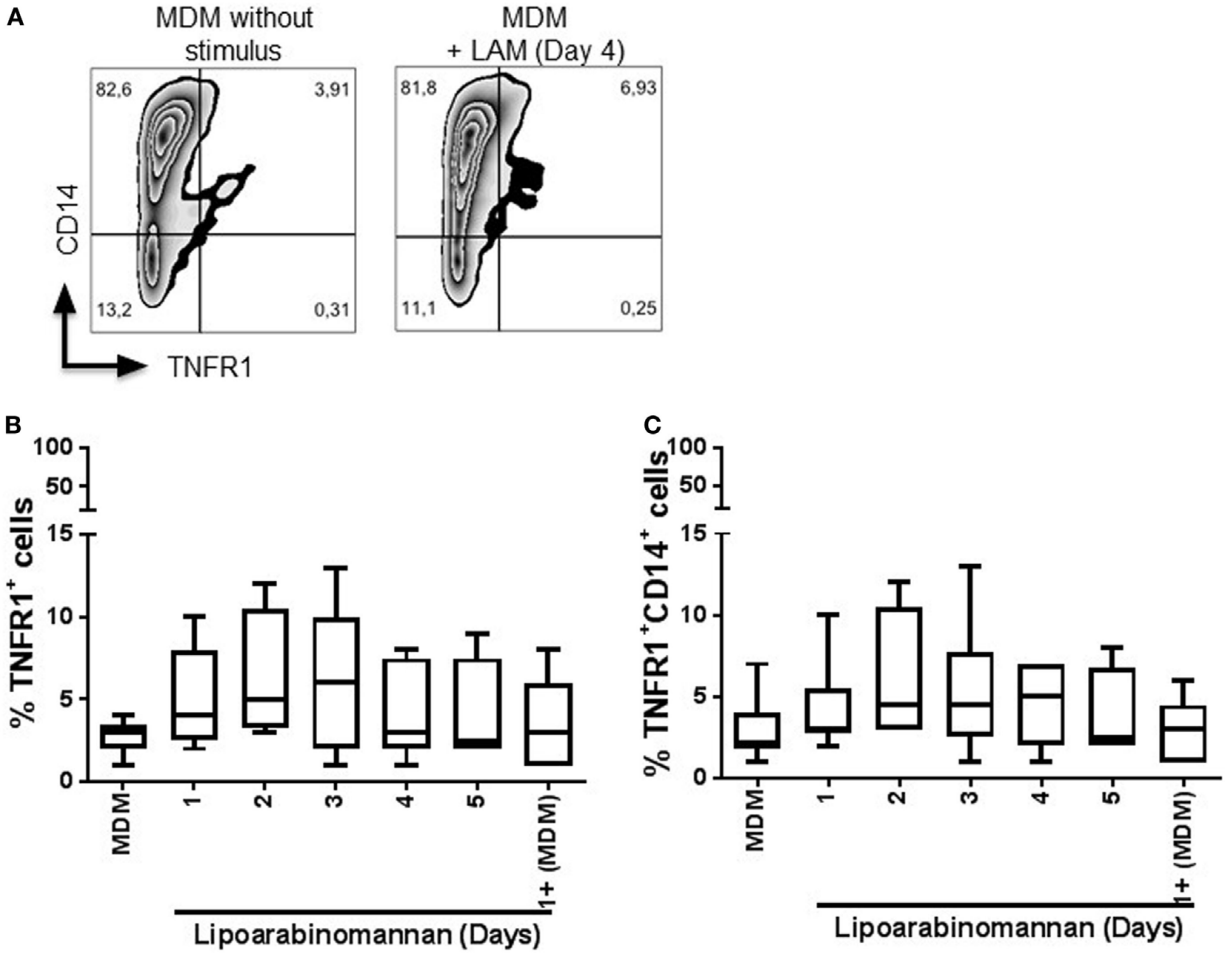
Frequency of tumor necrosis factor receptor 1 (TNFR1^+^) monocyte-derived macrophages (MDM) is not affected by lipoarabinomannan (LAM) exposure. Pure monocytes were exposed to LAM for 1, 2, 3, 4, or 5 days, and at day 7, cells were recovered to perform flow cytometry analyses. Representative zebra plot of MDM unexposed or exposed to LAM for 4 days (left and right, respectively) **(A)**. Flow cytometry analysis of TNFR1 independent or dependent of CD14 co-expression **(B,C)**. Data are representative of five independent experiments. Box plot indicates median ± interquartile range (5–95).

**Figure 7:**
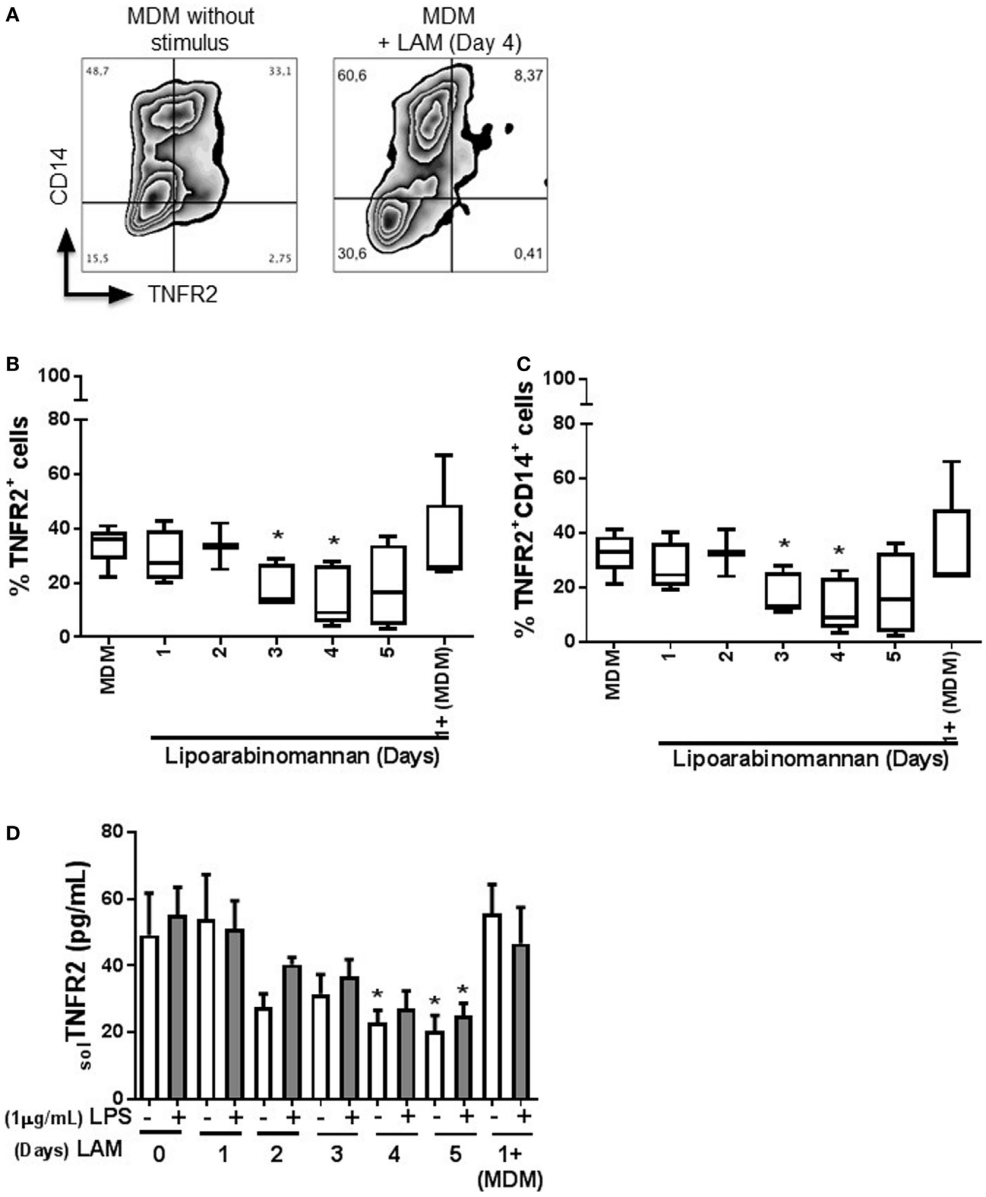
Frequency of tumor necrosis factor receptor 2 (TNFR2^+^) monocyte-derived macrophages (MDM) and soluble TNFR2 are decreased when monocytes are exposed to lipoarabinomannan (LAM). Pure monocytes were exposed to LAM for 1, 2, 3, 4, or 5 days, and at day 7, cells were stimulated or not with LPS (1 µg/mL) for 24 h and then recovered at day 8. Flow cytometry zebra plot of MDM without stimulus or exposed for 4 days to LAM (left and right, respectively) **(A)**. Flow cytometry analysis of MDM expressing TNFR2 independent or dependent of CD14 co-expression **(B,C)**. Soluble form of TNFR2 was measured in supernatants by ELISA **(D)**. Data are representative of five independent experiments. Box plot indicates median ± interquartile range (5–95), and bars represent median ± SD. *P < 0.05, Kruskal–Wallis and Dunn’s *post hoc* tests compared to unexposed MDM.

Next, we evaluated the expression of TNFR1 and TNFR2 (Figures 6 and 7, respectively), to explore if LAM stimulus *per se* can affect the TNF pathway. We found that TNFR1 expression is not affected on MDM cell surface, independently of the length of exposure to LAM stimulus, and the frequency of TNFR1^+^ is not dependent of the CD14 co-expression (Figures 6B,C). In contrast, TNFR2 frequency is downregulated on MDM as a consequence of LAM exposure during a long time (Figures 7B,C). To validate our result, the solTNFR2 form was measured by ELISA. The LPS stimulus was included in order to verify if an activation process can potentiate the effect regarding TNFR2 expression similar to TNF. Our data confirmed that MDM exposed to LAM for 4–5 days released less solTNFR2 than MDM not exposed to LAM or exposed for a short time; however, those MDM maintained the same low level of solTNFR2 even after stimulation with LPS (Figure 7D).

### PARP and JNK Pathways Are Not Affected in Mature Macrophages When Monocytes Are Exposed to LAM

**Figure 8:**
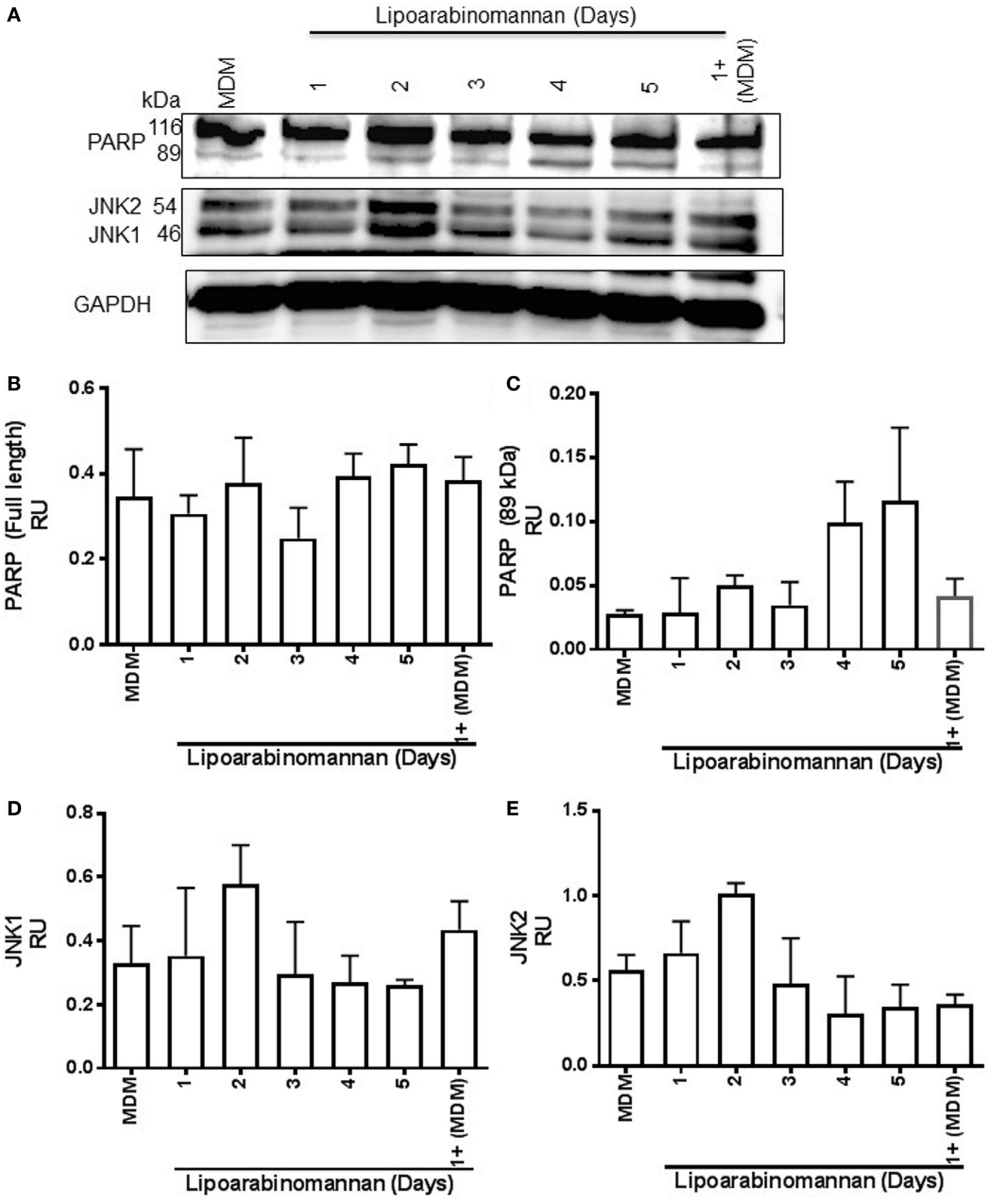
Poly(ADP-ribose) polymerase (PARP) and Jun N-terminal kinase (JNK) pathways are not affected when monocytes are exposed to lipoarabinomannan (LAM). Pure monocytes were exposed to LAM for 1, 2, 3, 4, or 5 days, and at day 7, cells were recovered to perform Western blot assays. Representative Western blot of monocyte-derived macrophages (MDM) lysates for PARP, JNK, and glyceraldehyde 3-phosphate dehydrogenase (GAPDH) which was used as a loading control **(A)**. Band densities of full-length and fragmented PARP (116 and 89 kDa, respectively) **(B,C)** and JNK1 and JNK2 forms (46 and 54 kDa, respectively) **(D,E)** were normalized with GAPDH by densitometry. Results are shown in relative units of concentration using the ImageJ 1.39c software. Data are representative of two independent experiments. Each bar indicates mean ± SD.

The interaction of TNF with either TNFR1 or TNFR2 induces a different activation pathway, even if both receptors can activate the canonical NF-κB pathway and the JNK MAP kinase pathway. TNFR1 generates an anti-apoptotic and pro-inflammatory response; however, TNFR2 also can activate the no-canonical NF-κB pathway to induce differentiation and survival process (20). PARP is a nuclear protein that can regulate key cellular process such as DNA repair and cell death. It has been described that TNF is involved in caspase-8 and PARP activations, and JNK1 is required for PARP-induced mitochondrial dysfunction (21, 22). To verify if TNF activation by LAM stimulus (4–5 days) affects PARP, JNK1, and JNK2 pathways to induce cell damage, these molecules were evaluated by western blot assay (Figure 8A). The detected full-length or fragment PARP (116 and 89 kDa, respectively) were not affected on MDM derived from monocytes exposed for short or long time to LAM (Figure 8B). We observed that the PARP cleaved fragment (89 kDa) was present in a double concentration in MDM exposed for a long time to LAM (96–120 h) compared to MDM not exposed to LAM, but these difference were not statistically significant (Figure 8C). Regarding JNK1 and JNK2, they were increased twice on MDM derived from 2 days LAM-exposed monocytes in comparison to MDM generated in absence of LAM stimulus; but this was not statistical different (Figures 8D,E). These data suggest that although LAM-exposed MDM affects TNF and TNFR2, the PARP and JNK pathways are not affected.

### LAM Stimulus Affects PAR2 Level and the Ability to Control *Mtb* Intracellular Growth

**Figure 9:**
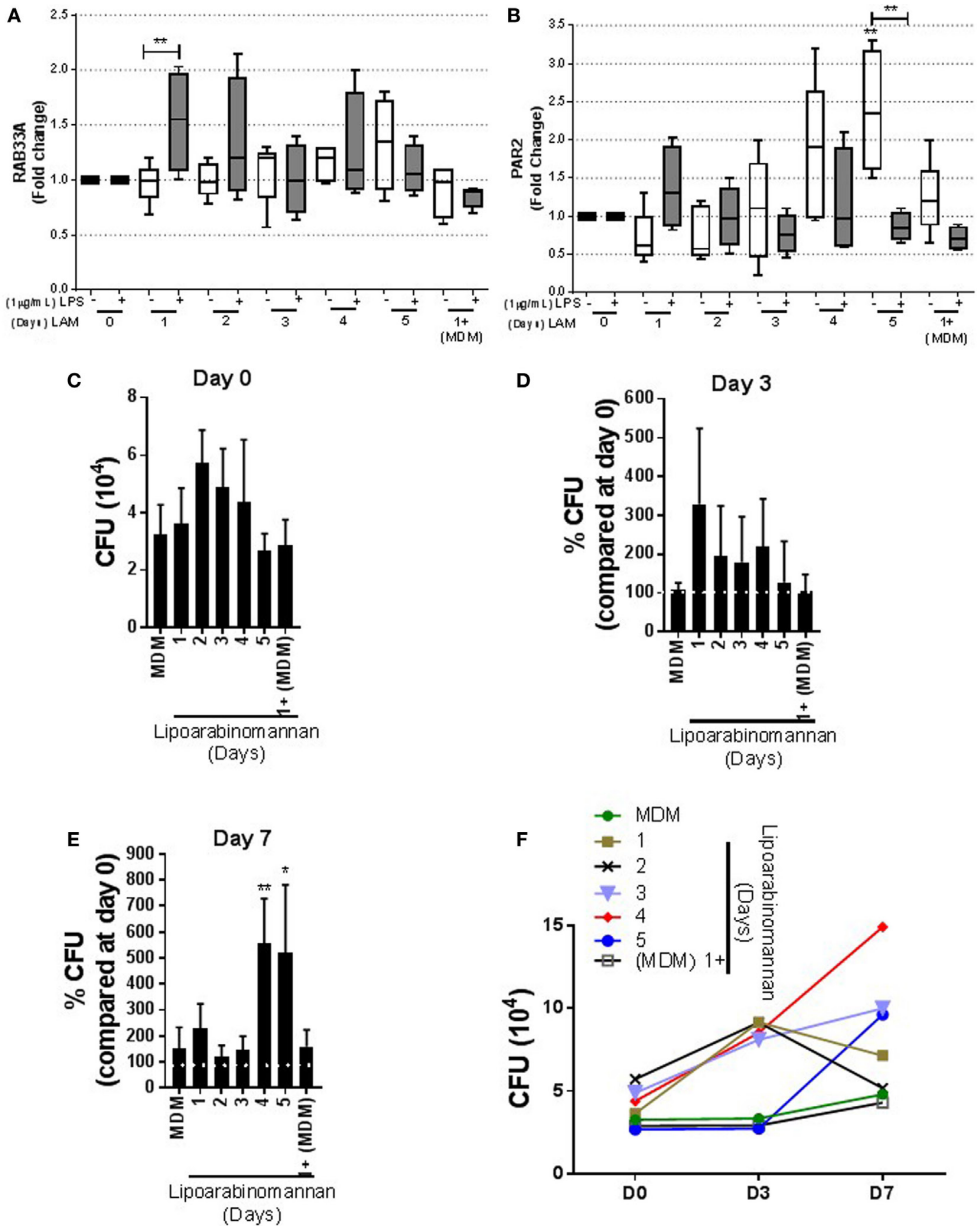
Proteinase-activated receptor-2 (PAR2) production and bacterial growth are affected in monocyte-derived macrophages (MDM) when monocytes are exposed to lipoarabinomannan (LAM) for long time. Pure monocytes were exposed to LAM for 1, 2, 3, 4, or 5 days, and at day 7, cells were infected with *Mycobacterium tuberculosis* (*Mtb*)-H37Ra (MOI 1) or cells were stimulated or not with LPS (1 µg/mL) for 24 h and at day 8 recovered for RT-PCR. RAB33A and PAR2 relative expressions were measured by real-time PCR [**(A,B)**, respectively]. Colony-forming unit (CFU) assay measuring living *Mtb*-H37Ra bacteria in infected MDM on days 0, 3, and 7 post-infection. Day 0 was considered the basal living *Mtb*-H37Ra that has been engulfed by MDM **(C)**. Using as 100% the bacteria uptake at day 0 per individual experimental condition (white line), the percentage of CFU change was calculated at days 3 and 7 [**(D,E)**, respectively]. Summary of absolute number of CFU counted at days 0, 3, and 7 post-infection **(F)**. Data are representative of 4–5 independent experiments. Box plot indicates median ± interquartile range (5–95), and bars indicate mean ± SD. *P < 0.05, **P < 0.01. Kruskal–Wallis and Dunn’s *post hoc* tests, MDM exposed to LAM were compared to unexposed MDM.

To investigate whether LAM stimulus on MDM affects the presence of molecules necessary to control *Mtb* intracellular growth, we evaluated the presence of RAB33A, a small GTP-binding protein whose transcription is reduced in active TB (23). Our data showed that RAB33A transcription level is not affected in MDM when monocytes were exposed to LAM, although 24 h LAM-exposed monocyte MDM were activated by LPS and able to produce higher levels of RAB33A compared with not activated MDM (Figure 9A). We also measured the PAR2, a receptor activated by serine proteinases. It has been reported that PAR2 activation alters the macrophage phenotype toward a pro-inflammatory-like profile associated with high levels of TNF (24). PAR2 transcriptional level was increased in MDM when monocytes were exposed for a long time (4–5 days) in comparison with MDM without LAM stimulus. Interestingly, when MDM under a long-time exposure to LAM were activated with LPS, PAR2 gene expression was decreased (Figure 9B).

Finally, we evaluated the anti-microbial activity of MDM exposed to LAM. We infected MDM with *Mtb*-H37Ra at MOI 1, and CFU was evaluated at days 0, 3, and 7 post-infection.To evaluate bacterial intracellular growth, the percentage of CFU in relation to the number of bacteria engulfed by MDM generated under different LAM exposure times was obtained. The percentage of CFU was calculated by considering CFU at day 0 (CFU_day 0_) per individual experimental condition (Figure 9C) as 100% CFU in relation to CFU at day 3 (CFU_day 3_) and day 5 (CFU_day 5_) post-infection. We found that MDM with a short- or long-time exposure to LAM maintained similar ability of mycobacterial phagocytosis, even if MDM generated under LAM stimuli for 48–96 h showed increased CFU, but this was not statistically different at day 0 (Figure 9C). CFU measured at day 3 post-infection showed similar CFU counts for MDM generated under LAM stimulus or not exposed to LAM (Figure 9D); however, MDM exposed during differentiation to LAM for a longer time (4–5 days) showed a strong increase of CFU count at day 7 post-infection (Figure 9E). Using the absolute number of CFU also confirmed the increased of CFU at day 7 post-infection and showed that MDN exposed for 4–5 days to LAM contained the highest CFU at day 7 post-infection (Figure 9F). Together, these data suggest that longer LAM stimulus results in an increased expression of molecules such as PAR2 to activate anti-mycobacterial pathways. However, MDM whose monocytes were exposed to LAM for long time were not able to respond to activation stimulus such as LPS and mycobacteria. This suggests that these MDM are not efficient to control *Mtb* intracellular growth.

## Discussion

In this study, we found that LAM exposure of monocytes during differentiation toward macrophages modifies the expression of GAL9 and leads to MDM with reduced capacity to respond to an inflammatory stimulus and to eliminate intracellular mycobacteria. Our previous studies have shown that maturation and expression of Toll-like receptors are altered in MDM generated under LAM exposure (9). We now show that MDM dysfunction caused by LAM exposure affects TNF and PAR2 pathways and the control of intracellular mycobacterial growth.

Previous reports have shown that the expression of GAL9 and TIM3 molecules was downregulated during an *Mtb* infection and that GAL9/TIM3 pathway was necessary to activate bactericidal mechanisms to control intracellular bacterial growth (11, 12). However, it was unknown if the decrease of GAL9 and TIM3 expression on MDM surface could be induced only by the infection with the whole mycobacteria or if only LAM exposure could also affect the expression of these molecules. We postulated that LAM, which is an important virulent factor of *Mtb*, could affect the control of intracellular bacterial replication and explored at the molecular level several molecules involved in this process. Indeed, monocytes are in contact with mycobacterial glycolipids in the blood circulation and this can affect their maturation toward functional macrophages (5, 9). In fact, it is known that LAM can incorporate itself into the membrane of T cells to inhibit phosphorylation of TCR-dependent molecules in order to inhibit T-cell activation (25, 26). However, the molecular mechanisms affecting the macrophage differentiation are not defined. In addition, as it can be expected, LAM exposure of monocytes can modify molecules that are involved in the control of microbial immunity in macrophages such as the TNF pathway (11, 19).

Our results show that TIM3 expression is not affected by LAM exposure during MDM differentiation. It has been shown that patients with pulmonary TB have low numbers of TIM3^+^ monocytes in peripheral blood, and using an *in vitro* infection model, it has been observed that both pathogenic and no pathogenic strains of *Mtb* decreased the frequency of MDM TIM3^+^ and GAL9^+^ MDM (12). It is possible that TIM3 expression on MDM could be regulated trough a more complex signal than that induced by only one glycolipid such as LAM. Another possibility could be that LAM stimulus induces a downregulation of TLR molecules on the cell surface, and consequently, TIM3 expression is not changed. Previous reports have shown that TLR-dependent activation induces a significant reduction of TIM3 expression in the macrophage (27).

We then studied GAL9 expression, one of the best studied natural ligands of TIM3, and observed that it was clearly downregulated by LAM stimulus independently of CD14 co-expression on MDM. We noticed that monocytes required long exposure time (4–5 days) with LAM to generate a low frequency of GAL9^+^ MDM. GAL9 regulation was also affected at the transcriptional level, and consequentially, the solGAL9 form was decreased. Thus, even if *Mtb* infection decreases both GAL9 and TIM3 expressions, it is now clear that the mechanism regulating their expression is different from exposure to LAM only, as LAM stimulus was enough to decrease GAL9 levels but not TIM3. It has been reported that the role of monocyte-derived GAL9 is important to induce the activation of other cells such as natural killer cells (28). A study analyzing GAL9 has proposed that plasma levels could reflect the status of inflammation and disease severity in malaria infection (29). Therefore, it is possible that this downregulation of GAL9 is used by *Mtb* as first step to manipulate the activation of pro-inflammatory responses in macrophage.

Jayaraman et al. have reported that GAL9/TIM3 pathways induced IL-1β, which allows downstream TNF production and therefore caspase-dependent restriction of intracellular bacterial growth (19). Based on these data, these pathways were evaluated in the present study. We found that the frequency of IL-1R^+^ MDM was not modified by LAM stimulus, which is in agreement with previous data suggesting that viable *Mtb* total bacteria are required to induce IL-1β secretion by macrophage (30). However, when TNF was measured, our data showed that the frequency of MDM expressing tmTNF was increased after monocyte exposure to LAM for 4–5 days. We also found that TNF was upregulated in MDM exposed to LAM at the transcriptional level and as previously reported, the presence of solTNF was decreased when LAM exposed MDM were activated with a second stimulus such as LPS (9). However, our results revealed that TACE expression, at the transcriptional level, was not affected in MDM generated under LAM stimulus. Our results confirmed that when LAM-exposed MDM received a stimulus (LPS), they were not able to produce TNF at the transcriptional and protein levels as we previously reported (9). An alternative hypothesis to explain TNF levels as consequence of LAM stimulus was that LAM exposure can also induce an increase of TNFR level. This hypothesis was discarded because the expression of TNFR1 was not modified and TNFR2 was decreased when monocytes were exposed 3–5 days to LAM. TNFR2 was downregulated on the cell surface as well as its soluble TNFR2. Furthermore, the soluble TNFR2 was still downregulated when MDM were activated by LPS. Together data show that LAM exposure of monocytes during MDM differentiation affects TNF pathway and subsequent responses of MDM can be impaired. TNF pathway is involved in anti-bacterial activity of human macrophages, which could be affected in LAM-exposed MDM.

Proteinase-activated receptor-2 has been shown to be a modulator of innate and adaptive immunity during infections, helping macrophage differentiation toward a pro-inflammatory phenotype, favoring mainly TNF production (24, 31). Our result showed that PAR2 has an expression pattern similar to TNF. The PAR2 gene expression was increased in MDM when monocytes were exposed for a longer time to LAM but when those MDM were again activated, PAR2 production was blocked. We cannot know if this downregulation is a consequence of the TNF downregulation or the contrary. Since long exposition to LAM generates MDM unable to produce TNF and PAR2 in response to LPS stimulus, we thought that LAM exposed MDM deficiencies may persist when other additional stimuli are encountered including mycobacterial infection. MDM exposed to LAM during their differentiation showed impaired capacity to control the intracellular growth of *Mtb*-H37Ra at day 7 post-infection. This shows that long-term LAM exposure is detrimental for the anti-microbial capacity of MDM resulting in the lack of control of intracellular bacterial growth.

**Figure 10:**
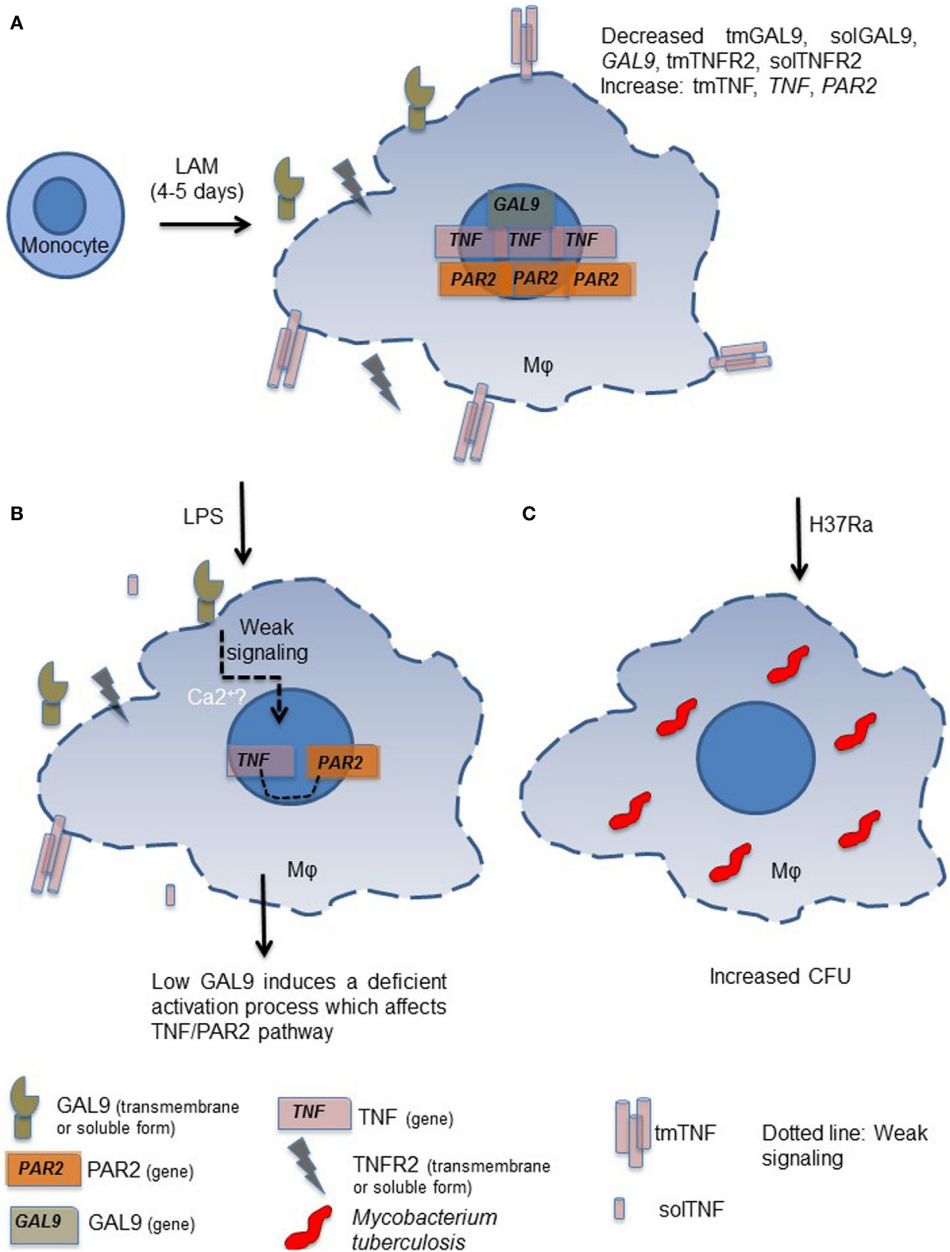
Proposed model to explain the inability to control intracellular *Mycobacterium tuberculosis* growth by monocyte-derived macrophages (MDM) generated under exposure to lipoarabinomannan (LAM). Monocytes exposed for long time to LAM (4–5 days) differentiate into MDM that are characterized by deficiency in galectin (GAL)9 and TNFR2 but high tumor necrosis factor (TNF) and proteinase-activated receptor-2 (PAR2) expressions **(A)**. When altered LAM-exposed MDM are in contact with a second stimulus such as LPS only week signal are triggered and consequently the activation of crucial pathways such as TNF and PAR2 are affected **(B)**. The GAL9/TNF/PAR2 axis is crucial for the activation of microbicidal mechanism necessary to eliminate intracellular mycobacteria, as it is expected. MDM generated under LAM exposure are not able to efficiently eliminate intracellular bacteria **(C)**.

In conclusion, our study shows that when monocytes are exposed for long time (4–5 days) to a microenvironment in which LAM is present, generated macrophages exhibit a different phenotype characterized by a decreased expression of GAL9 (Figure 10A). Although our knowledge of the signaling events triggered by GAL9 pathway on macrophages remains limited, we are aware that in human lymphocytes, this pathway increases cytosolic calcium (Ca^2+^) mobilization, which in turn is required for the production of pro-inflammatory cytokines (Figure 10B) (32). When LAM-exposed MDM are subsequently activated by other stimuli such as *Mtb* infection, they are not able to respond and eliminate intracellular bacterial as unexposed MDM (Figure 10C) (32).

## Author Contributions

LC-G conceived and designed the experiments. LR-L, CC, and LC-G performed the experiments. LR-L, IS-O, IG, and LC-G analyzed and discussed the data. CC and IS-O contributed reagents/materials/analysis tools. LR-L, IG, and LC-G wrote the paper.

## Conflict of Interest Statement

The authors declare that the research was conducted in the absence of any commercial or financial relationships that could be construed as a potential conflict of interest.
