# Supplementary figures and images for: Lipoarabinomannan Decreases Galectin-9 Expression and Tumor Necrosis Factor Pathway in Macrophages Favoring Mycobacterium tuberculosis Intracellular Growth
Source: Front Immunol. 2017 Nov 27;8:1659. doi: 10.3389/fimmu.2017.01659 (PMC5711832; doi:10.3389/fimmu.2017.01659)

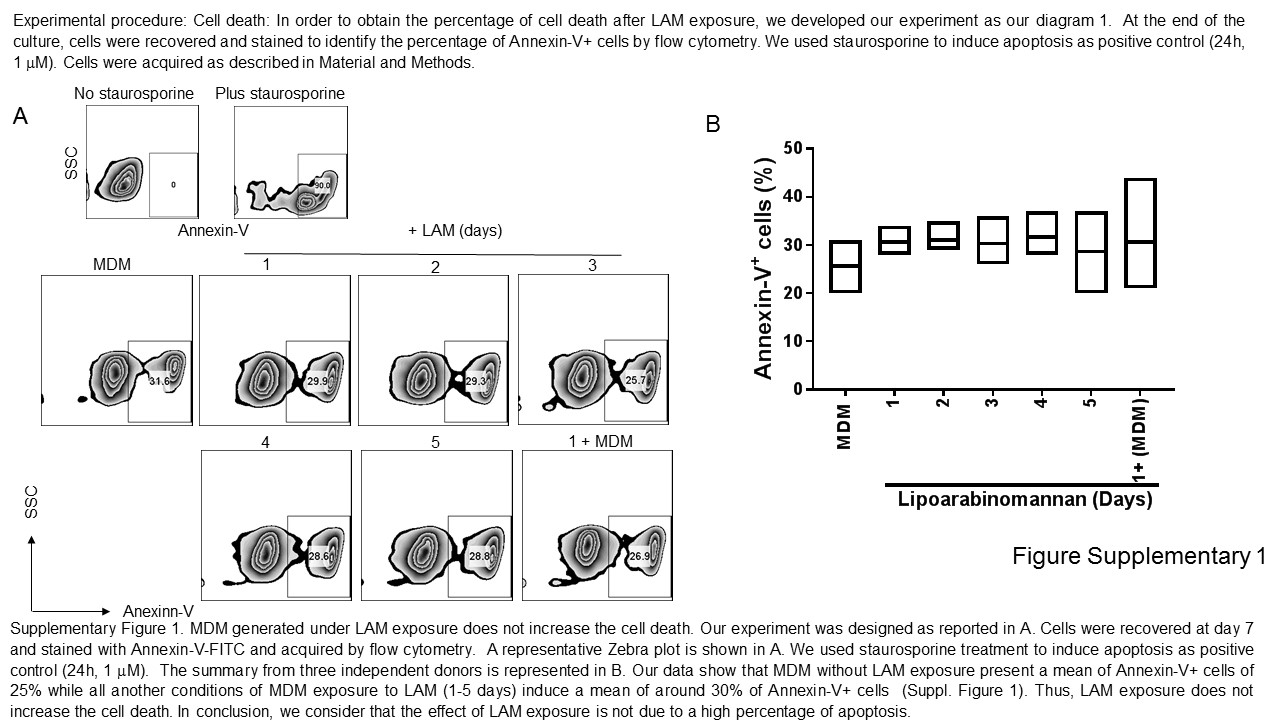

Supplement: Supplementary file 1 [file Image_1.JPEG]
